# PIC micro-controller based synchronization of two fractional order jerk systems

**DOI:** 10.1038/s41598-022-17029-x

**Published:** 2022-08-22

**Authors:** Samuel Tagne, Bertrand Bodo, Guy François V. Ayissi Eyebe, Jean Sire A. Eyebe Fouda

**Affiliations:** Physics, University of yaounde 1, Yaounde, 812 Cameroon

**Keywords:** Applied physics, Electronics, photonics and device physics

## Abstract

The paper studies a 3D Chaotic Jerk oscillator with fractional derivatives. An approach is proposed to implement it on a PIC16F877A microcontroller in order to reduce the requirements for multiple analogue electronic components such as resistors, capacitors, coils, multipliers, operational amplifiers, which are very bulky and consume a lot of power. The behaviours of the underlying system are analysed analytically, numerically and experimentally. It comes from this analysis that the fractional model exhibits chaotic dynamics when for parameters for which the equivalent integer derivative system exhibits limit-cycles. The synchronization under two closed initial conditions is also studied, highlighting one of the most common applications of the chaos concept.

## Introduction

Concept of fractional calculus has become undoubtdly a subjet of active research field in nonliear science, due to its potential applications in electronics^1^, mechanics^2^, nuclear physics^3^, medicine^4^, financial systems^5^. Likewise, chaos has been also widely reported over the past century years^6–13^. It is well-known that chaotic systems have a very high degree of sensitivity to initial conditions and their evolution through phase space that appears unpredictable.

In fact, in 1963, Lorenz emphasized that a chaotic system solved with two very close initial conditions could have two completely different dynamics^14^. More recently, some electronic circuits exhibiting chaotic behavior are proposed in literature^15–17^. Furthermore, those implementing fractional order circuit have been also reported. For example, the fractional Chen circuit^18,19^, the fractional Chua circuit^20^ and the fractional Rössler circuit^21^. For a better review one can read^22–24^.

In truth, fractional dynamical systems are developed with the main idea of introducing the memory effect in the dynamics of the system. It is then observed that these systems present hidden attractors that the conventional approach does not exhibit^25^. Considering this particular advantage, it is therefore imperative to design fractional electronic circuits able to reproduce the desired behavior. To do this, an ordinary capacitor is replaced by a fractional capacitor whose impedance value must be determined. In this case, the Laplace transform of the differential operator $\frac{1}{s}$ is replaced by $\frac{1}{s^m}$, where $0<m<1$ is the derivative order^26–31^.

However, to the best of our knowledge, it is arduous to determine the exact value of a fractional capacitor which corresponds exactly to his fractional derivative operator. Several techniques have been suggested to address this problem such as the Regular Newton’s Process^32^ and the Halley’s Iterative Method^33^. These methods were used to design the fractional circuits mentioned above using analogue component. From these circuits it can be seen that the analogue component approach requires very large capacitors and resistors which are difficult to find on the market. Furthermore, using large capacitors and large resistors means operating at high frequency^34^. We propose a numerical approach for the real implementation of a fractional system on a micro-controller based on Euler’s resolution method and applied to the synchronization of a Jerk system, which has never been done to our knowledge.

Jerk systems have important considerations for many applications in science and mechanical engineering. In^35^, it is noted that Jerk systems could exhibit several physical phenomena such as multi-stability, chaos or hyperchaos. They could be used for synchronization^36^ and encryption^37^ of chaotic systems.

In this paper, we propose an optimised algorithm to implement the fractional chaotic system in the numerical domain that is easy to prototype. The Jerk equation given in Refs.^38–40,42^ allow to achieve this. It will be useful to study the contribution of the fractional derivative on the dynamics of the system particularly if the implementation of the fractional system on a PIC-microcontroller allows to obtain hidden attractors contrary to the classical model considering the same parameters.

The paper is organised as follows: In the following section, we propose the description of the Jerk system followed by an analytical study. We explain the numerical methods dedicated to the computation of fractional integrals, we also present the method used for the implementation of the system on a micro-controller; “Results and discussions” is dedicated to the results and discussion; the paper ends with a conclusion and announces some perspectives for our future work.

## Mathematical models

### Background

In mechanics, a shake is a random change in the vector of acceleration without shock. In physics, the shock vector, more commonly called the jerk, is the acceleration vector’s derivative over time. Jerk systems are then the third-order differential equations of the form $\frac{d^3 x}{dt^3} = F ( x)$, which translates the variation of the acceleration in the system. It is the simplest of three-dimensional chaotic systems, where *F*(*x*) is the nonlinear function that describes the third-time derivative of displacement variable *x*. In this work, the Jerk system used will be the one presented in the Eq. (1)^40^.

1 $$\begin{aligned} \frac{d^3 x}{dt^3} =-\beta \frac{d^2 x}{dt^2}-\frac{d x}{dt}+\alpha x^3-x. \end{aligned}$$

$\alpha$ and $\beta$ could be subsequently defined as control parameters. This equation (1) can be transformed into the following system.

2 $$\begin{aligned} \left\{ \begin{array}{lll} \dot{x} &{}=&{}y\\ \dot{y} &{}=&{}z\\ \dot{z} &{}=&{}\alpha x^3-x-y-\beta z. \end{array}\right. \end{aligned}$$

To solve it numerically, one use the Euler algorithm. In fact, the Euler algorithm has only one step, and it is easy to implement because it requires fewer mathematical operations^41^. The Euler algorithm is described for Eq. (1) by:

3 $$\begin{aligned} \left\{ \begin{array}{lll} x_{n+1} = x_{n} + hy_{n}\\ y_{n+1} = y_{n} + h z_{n}\\ z_{n+1} = z_{n} + h (\alpha x_{n}^3- x_{n}-y_{n}-\beta z_{n} ) \end{array}\right. \end{aligned}$$

Physically, (*x*, *y*, *z*) respectively represents the position, the velocity and the acceleration (Eq. 1) could be dissipative if $\beta >0$. On can therefore highlight three equilibrium points, so $E_1(0,0,0)^T,\; E_2(0,0,-\frac{1}{\sqrt{\alpha }})^T$, and $E_2(0,0,\frac{1}{\sqrt{\alpha }})^T$. The Jacobian matrix of (Eq. 1) is given by

4 $$\begin{aligned} J=\left( \begin{array}{ccc} 0 &{} 1 &{} 0 \\ 0 &{} 0 &{} 1 \\ 3\alpha x^2-1 &{} -1 &{} -\beta . \end{array}\right) \end{aligned}$$

We come out two characteristics equations relating to $J_0, \;J_1$ and $J_2$

5 $$\begin{aligned} \begin{array}{c} \lambda ^3 +\beta \lambda ^2 + \lambda + 1=0\;\text{ and }\; \lambda ^3 + \beta \lambda ^2 + \lambda - 2=0. \end{array} \end{aligned}$$

The literature shows that the Routh–Hurwitz criterion contains the necessary and sufficient conditions for the system stability. Thus, without even solving the characteristic equation, one finds that the system is stable for $\beta >0$ whatever the chosen equation.

### Route to chaos

It is shown in Refs.^42–47^ that the nature of the chaotic dynamic system, in addition to being sensitive to initial conditions, is closely linked to another parameter which is called the control parameter. In our case, this is the $\beta$ parameter. Thus, the system will behave chaotic depending on this parameter.

**Figure 1:**
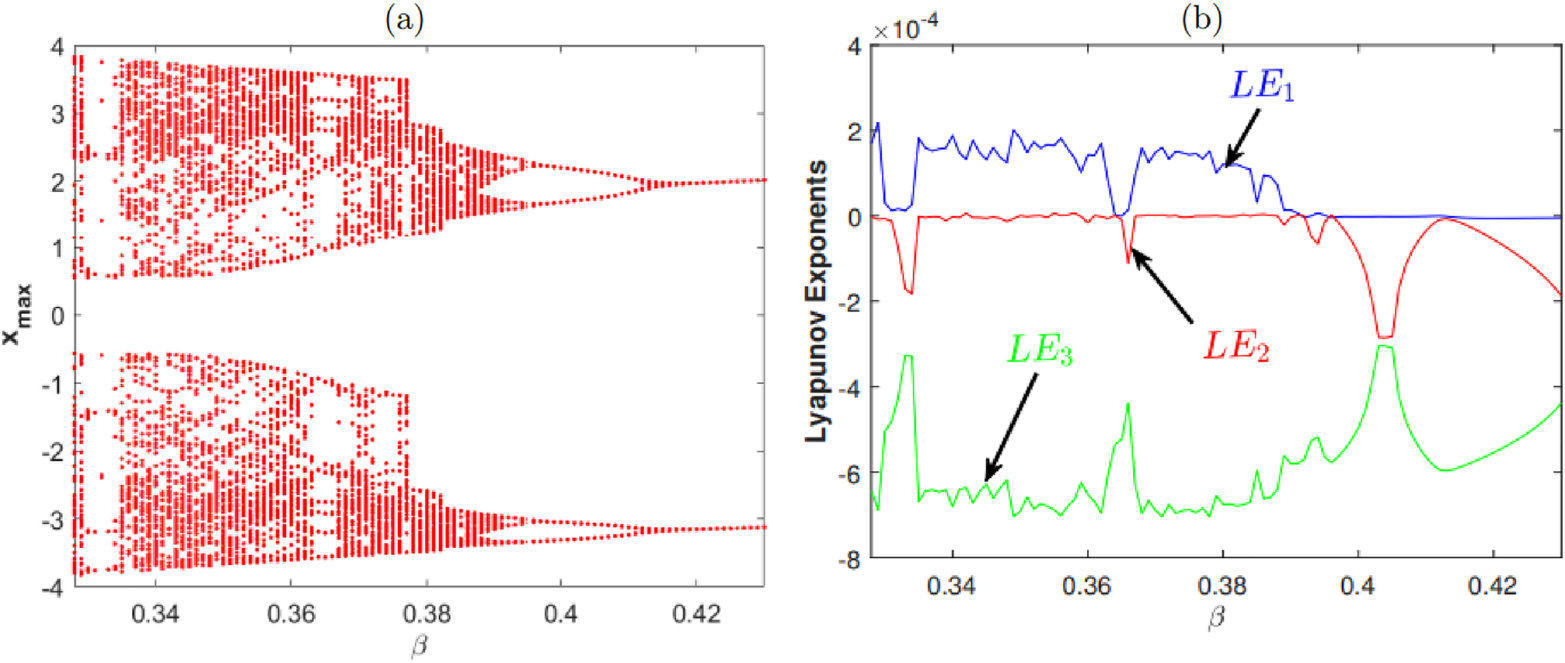
Bifurcation diagram and Lyapunov exponents for $\beta \in [0.328,0.43]$, $\alpha =\frac{1}{10}$, under initial conditions: $(x_0,y_0,z_0)=(0,1,0)$.

We report in Fig. 1a the bifurcation diagram of the system. The diagram shows a high concentration of points corresponding to the system dynamics change for $0.33<\beta <0.363$ and $0.371<\beta <0.378$. Over these intervals, the system changes periodically and it is therefore difficult to observe chaotic dynamics. In Fig. 1b, the lyapunov exponents are plotted against $\beta$. Indeed, Lyapunov exponents obtained using Wolf’s algorithm are another tool used to decipher the nature of a dynamical system^48–53^. Therefore, presence of positive Lyapunov exponents ($\lambda _1$) is sufficient to establish that the considered system is able to exhibit chaotic dynamics. Moreover, the Lyapunov Exponents considered against the variation of the system’s control parameter show a large superposition with the bifurcation diagram as illustrated in the Fig. 1b. When $\beta =0.35$, one obtain after 1000 iteration by step of 0.01, $\lambda _1= 0.089577,$ $\lambda _2= 0.001118 \text{ and } \,\lambda _3= -0.447794$. The Kaplan–Yorke dimension of the system is $D_L =2.2025$. Thus according to Ref.^57^, this Jerk system generates chaotic behaviours.

### Fractional model

There are several definitions of the fractional order derivative in the literature, but the fractional Caputo and Riemann–Liouville (R–L) operators are the most commonly used in different areas of fractional dynamical systems. The main advantage of the fractional Caputo derivative over the fractional R–L derivative is that the initial conditions of fractional differential equations with Caputo derivatives are the same as those of the integer order for differential equations^53^. Therefore, in this paper we will use the initial conditions $(x_0,y_0,z_0)=(0,1,0)$ to solve the integer derivative and fractional derivative system. Fractional calculus is a generalization of integration and differentiation to noninteger-order fundamental operator $aD_t^q f(t)$^53^, where *a* and *t* are the bounds of the operation. The definition of the fractional derivative under Caputo for a function *f*(*t*) of *q* order is defined as follows^54^

6 $$\begin{aligned} aD_t^q f(t)=\left. \begin{array}{rr} \frac{1}{\Gamma (k-q)}\int _a^t\frac{f^k(\tau )}{(t-\tau )^{q-k+1}}d\tau \end{array}\right. \end{aligned}$$

for $n-1< q < n$ , $\Gamma (.)$ is Euler’s *Gamma* function.

7 $$\begin{aligned} \Gamma (q)=\int _0^\infty e^{-t}t^{q-1}dt. \end{aligned}$$

Thus, system (Eq. 1) can be rewritten as (Eq. 8) to obtain the fractional Jerk system.

8 $$\begin{aligned} \left\{ \begin{array}{lll} _0D_t^{q_1}x(t) &{}=&{}y(t)\\ _0D_t^{q_2}y(t) &{}=&{}z(t)\\ _0D_t^{q_3}z(t)&{}=&{}\alpha x^3(t)-x(t)-y(t)-\beta z(t). \end{array}\right. \end{aligned}$$

### Numerical methods for calculation of fractional integrals

Hoda et al. have shown that by using Euler’s method, it is possible to find numerical solutions of linear and nonlinear systems of fractional differential equations. To prove this, they consider fractional derivatives as defined by Caputo. Furthermore, they show that Euler’s algorithm is very simple to implement and provides directly the solutions without linearization. Some examples illustrating numerical comparisons between the Euler algorithm and the classical algorithm are presented in Ref.^41^ to find the solution to a given dynamical system.

Let us consider the following problem: $D^qy(t)=f(t,y(t)),y(0)=y_0$, $0<q<1,t>0$. To solve it in the interval [0, *a*], it is necessary to construct a set of points $(t_j,y(t)),y(0)=y_0$ which are considered as approximate values of the solution. In order to perform this approximation, the interval [0, *a*] is divided into *n* sub-intervals $[t_j,t_{j+1}]$, each having an equal width. So, the general formula of the fractional Euler method is the following.

9 $$\begin{aligned} y(t_{j+1})= y(t_j)+\frac{h^q}{\Gamma (q+1)}f(t_j,y(t_j)), \end{aligned}$$

10 $$\begin{aligned} t_{j+1}= t_j+h,\qquad j=0,1,\ldots ,n-1. \end{aligned}$$

Observe that *y*(*t*) is an implicit system variable, the trapezoidal method is used to find it^55,56^. This method which consists in solving the system in two steps is called the prediction–correction approach that we use for and we obtain the following systems.

11 $$\begin{aligned} \left\{ \begin{array}{ll} y_p(t_k)=y(t_{k-1})+\frac{h^q}{\Gamma (q+1)}f(t_{k-1},y(t_{k-1})) \Longrightarrow {{Prediction}} \\ y(t_k)=\frac{qh^q}{\Gamma (q+2)}f(t_{k-1},y(t_{k-1}))+\frac{h^q}{\Gamma (q+2)}f(t_k,y_p(t_k))+y(t_k) \Longrightarrow {{Correction}} \end{array}\right. \end{aligned}$$

Applying this algorithm (Eq. 11) to the system (8), the solution is found in two steps as follows

12 $$\begin{aligned}&\left\{ \begin{array}{ll} \qquad \qquad \qquad \qquad {{Prediction system}} \\ \\ { x_p} = {x(k-1)}+{\frac{{h}^{{q_1}}}{\Gamma \left( { q_1+1} \right) }}{ y(k-1)} \\ y_p = y(k-1)+ \frac{h^{q_2}}{\Gamma (q_2+1)}z(k-1)\\ z_p = z(k-1) + \frac{h^{q_3}}{\Gamma (q_3+1)}\left[ \alpha x(k-1)^3 - x(k-1) -y(k-1)-\beta z(k-1)\right] \end{array}\right. \end{aligned}$$

13 $$\begin{aligned}&\left\{ \begin{array}{lc} \qquad \qquad \qquad \qquad {{Correction system}} \\ \\ x(k) =x(k-1)+ q_1 \frac{h^{q_1}}{\Gamma (q_1+2)}y(k-1) + q_1 \frac{h^{q_1}}{\Gamma (q_1+2)}y_p\\ y(k) =y(k-1)+ \frac{q_2h^{q_2}}{\Gamma (q_2+2)}z(k-1) + \frac{q_2h^{q_2}}{\Gamma (q_2+2)}z_p\\ z(k) =z(k-1)+ \frac{q_3h^{q_3}}{\Gamma (q_3+2)} \left[ \alpha x(k-1)^3- x(k-1) -y(k-1)-\beta z(k-1) \right] \\ + \frac{h^{q_3}}{\Gamma (q_3+2)}\left[ \alpha x_p^3- x_p-y_p\beta z_p\right] \end{array}\right. \end{aligned}$$

## Results and discussions

In this section, the methodology developed in the previous section is applied on the Jerk system to observe the impact of the fractional order on the resolution result. Although the implementation is independent of the discussion, we visualise here at the same time the theoretical synchronisation analyse under Matlab and experimental under micro-controller in order to compare them.

### Numerical analysis

**Figure 2:**
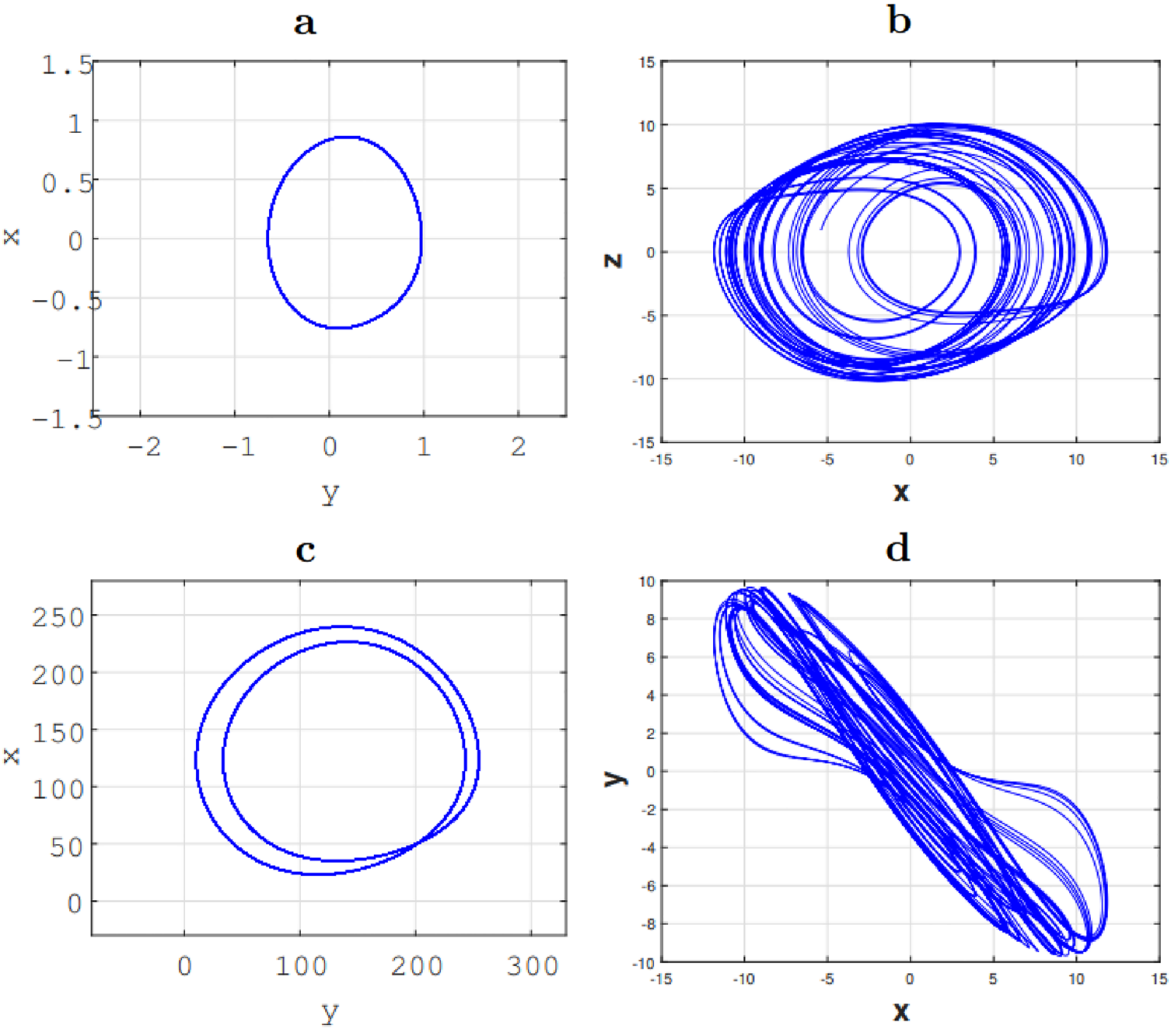
Phase portrait of the Fractional Jerk system showing route to chaos attractor when varied parameter $\beta$ (I): in (**a**) period-1 limit cycle for $\beta =0.4$, (**b**) a chaotic attractor for $\beta =0.35$. a and b are obtained with $(q_1,q_2,q_3)=(1,1,1)$. (II): in (**c**) a period-2 limit cycle attractor for $\beta =0.4$ obtained with $(q_1,q_2,q_3)=(0.96,0.97,0.97)$. (**d**) A chaotic attractor for $\beta =0.40$, obtained with $(q_1,q_2,q_3)=(0.98,1,1)$ under the initial condition $(x,y,z)=(0,1,0)$.

**Figure 3:**
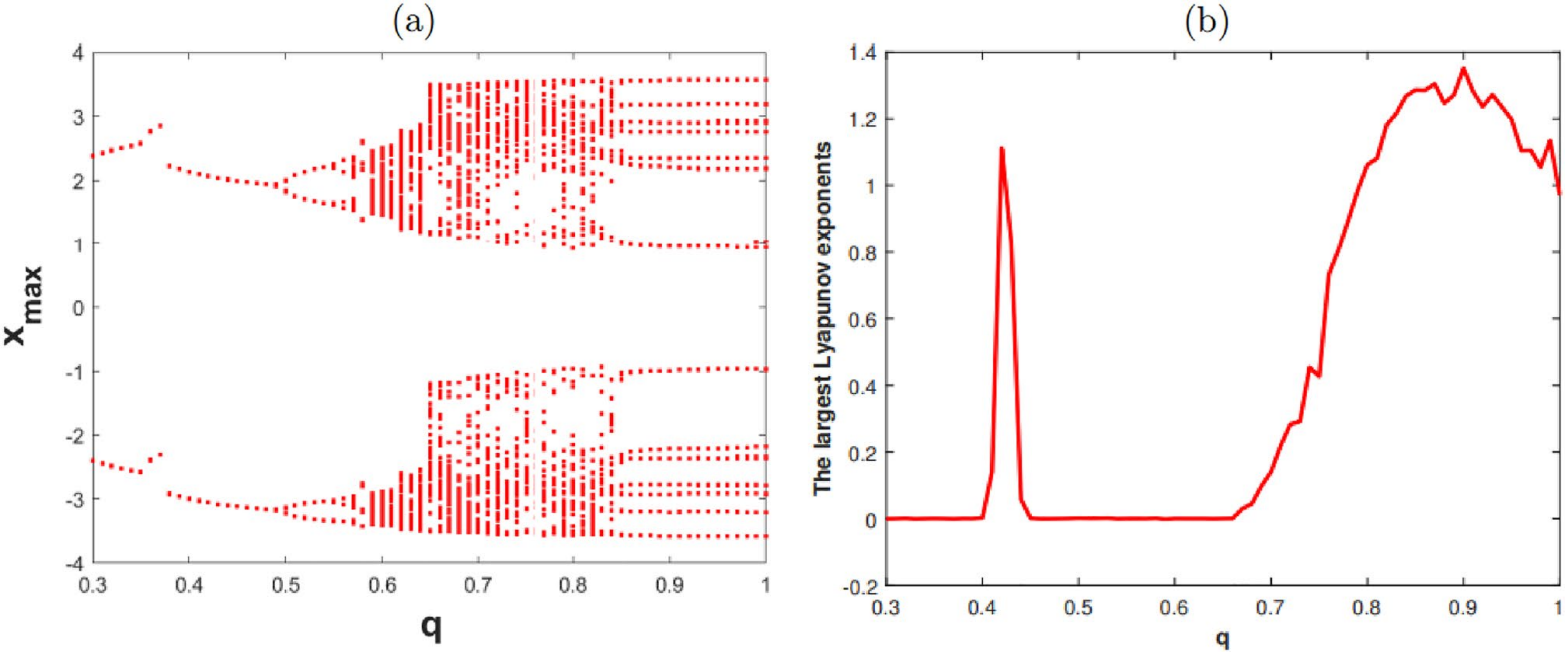
(**a**) Bifurcation diagram, (**b**) the largest Lyapunov exponents for $q=q_1=q_2=q_3$, $0.3<q<1$, $\beta =0.40$, $\alpha =\frac{1}{10}$ under initial conditions: $(x_0 , y_0 , z_0 ) = (0, 1, 0)$.

We implemented Eqs. (12) and (13) under the Matlab software, and one observed the attractors that we present in Fig. 2. On this figure, we have several main remarks to draw. From first view, the bifurcation diagram presented in the Fig. 1 shows that the system presents a periodic dynamics for $\beta =0.4$ and a chaotic dynamics for $\beta =0.35$. Under the assumption $q_1=q_2=q_3=1$, we observe for the two values of $\beta$ mentioned, a figure of periodic dynamics, Fig. 3a and a figure of chaotic dynamics, Fig. 2b respectively. This setting ($q_1=q_2=q_3=1$) is the equivalent of the classical resolute system. To prove the impact of the fractional order on the problem solution, let us consider the value of $\beta$ for which periodic dynamics are observed ($\beta =0.4$) and let us varies the fractional orders. In this way, taking $(q_1,q_2,q_3)=(0.94,0.98,0.95)$, Fig. 2c shows a double periodic dynamics. Still with ($\beta =0.40$) and considering $(q_1,q_2,q_3)=(0.98,1,1)$, one observes chaotic dynamics now (Fig. 2d), which was not observed for the classical case by considering ($q_1=q_2=q_3=1$), hence the interest of the fractional approach.

### Largest lyapunov exponent in the fractional model

Attractor presented in Fig. 2b is a distinctive sign that the fractional system has a chaotic behaviour, but it would be interesting to carry out investigations to quantify this chaotic behaviour. To do this, we use the Lyapunov exponent applied to time series^60^. Indeed, the fraction system does not return the easy task to calculate the system’s Jacobian matrix. Thus, we consider a state vector of the system and we reconstruct the phase space. This method allows us to calculate the largest Lyapunov exponent, which gives us $LLE=1.43>$0, and shows that the system is also chaotic in fractional model. The influence of the fractional order on the system can thus be represented in a general way. For this purpose, we present in Fig. 3a the bifurcation diagram and in Fig. 3b the spectrum showing the evolution of the largest lyapunov exponent according to the fractional order *q*. The superposition observed between these two figures shows that the system dynamics is indeed influenced by the fractional order *q*.

### Micro-controller implementation

**Figure 4:**
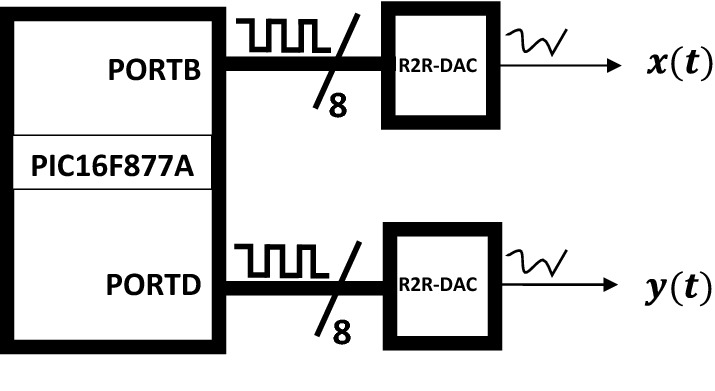
Schematic bloc for $x(t)-y(t)$ variable.

The Jerk system generates real continuous values, which are not understandable by the micro-controller. In order to solve this problem, we proposed here a shift of reference frame, which allows to switch from the analogue to the digital domain. Therefore, to digitise the analogue vector *x*, it is necessary to know the minimum and maximum values of *x*, the minimum and maximum reference values of the micro-controller. This implies knowledge of the precise number of bits on which to encode the converted values. Subsequently, a linear approximation line $X(x)=ax+b$ is defined, allowing to leave the interval $[x_{min},\; x_{max}]$ to $[X_{min},\;X_{max}]$. Here, $X_{min}=0$, $X_{max}=(2^n-1)$ and the number of bits is $n=8$. So $a=\frac{(2^n-1)}{x_{max}-x_{min}}$ and $b=(2^n-1)x_{min}$. In PICF877A micro-controller, system (3) is implemented via the Microchip XC8 compiler, the *x*(*k*) and *y*(*k*) variables was directed to the PORTB and PORTD and converted to an analogue voltage by the R-2R DAC as depicted on Fig. 4.

**Figure 5:**
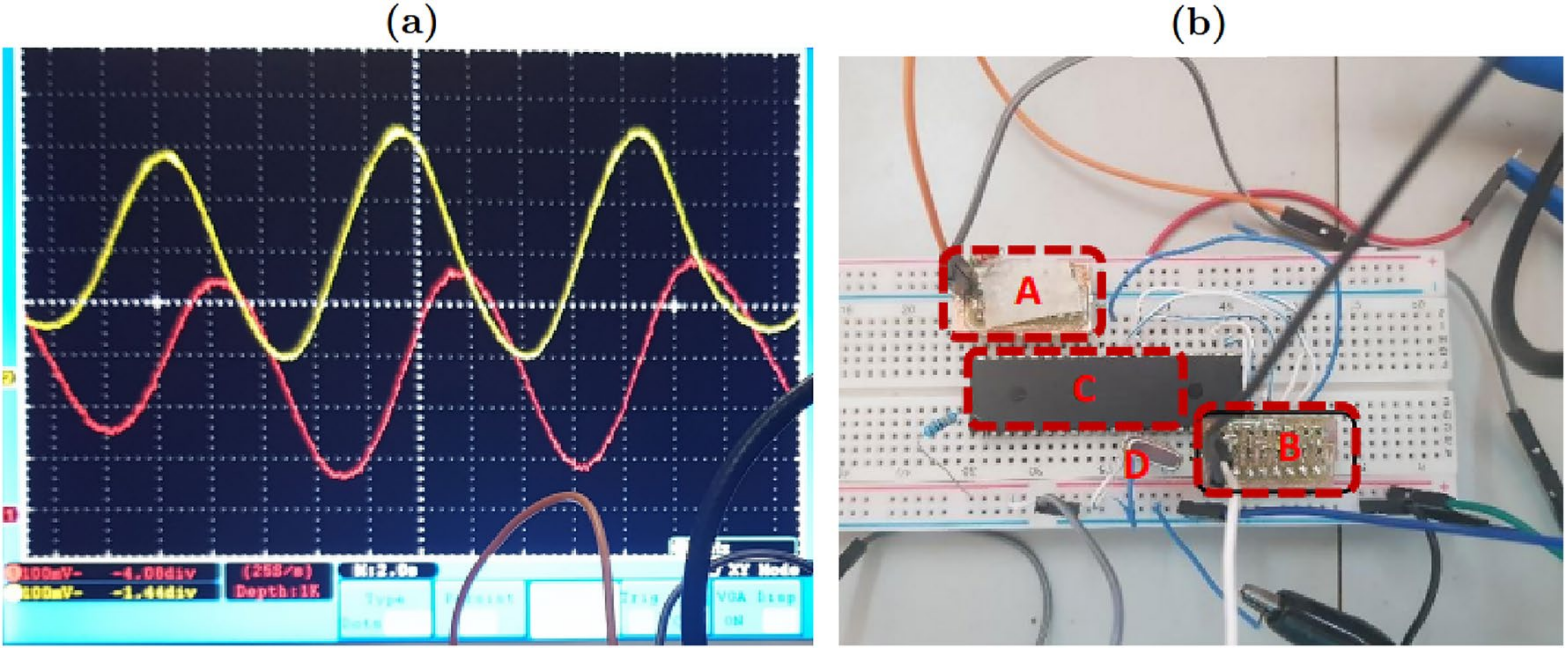
The digital circuit hardware platform implemented for the fractional Jerk system (**b**) and the induced time evolution acquired by the digital oscilloscope (**a**).

**Figure 6:**
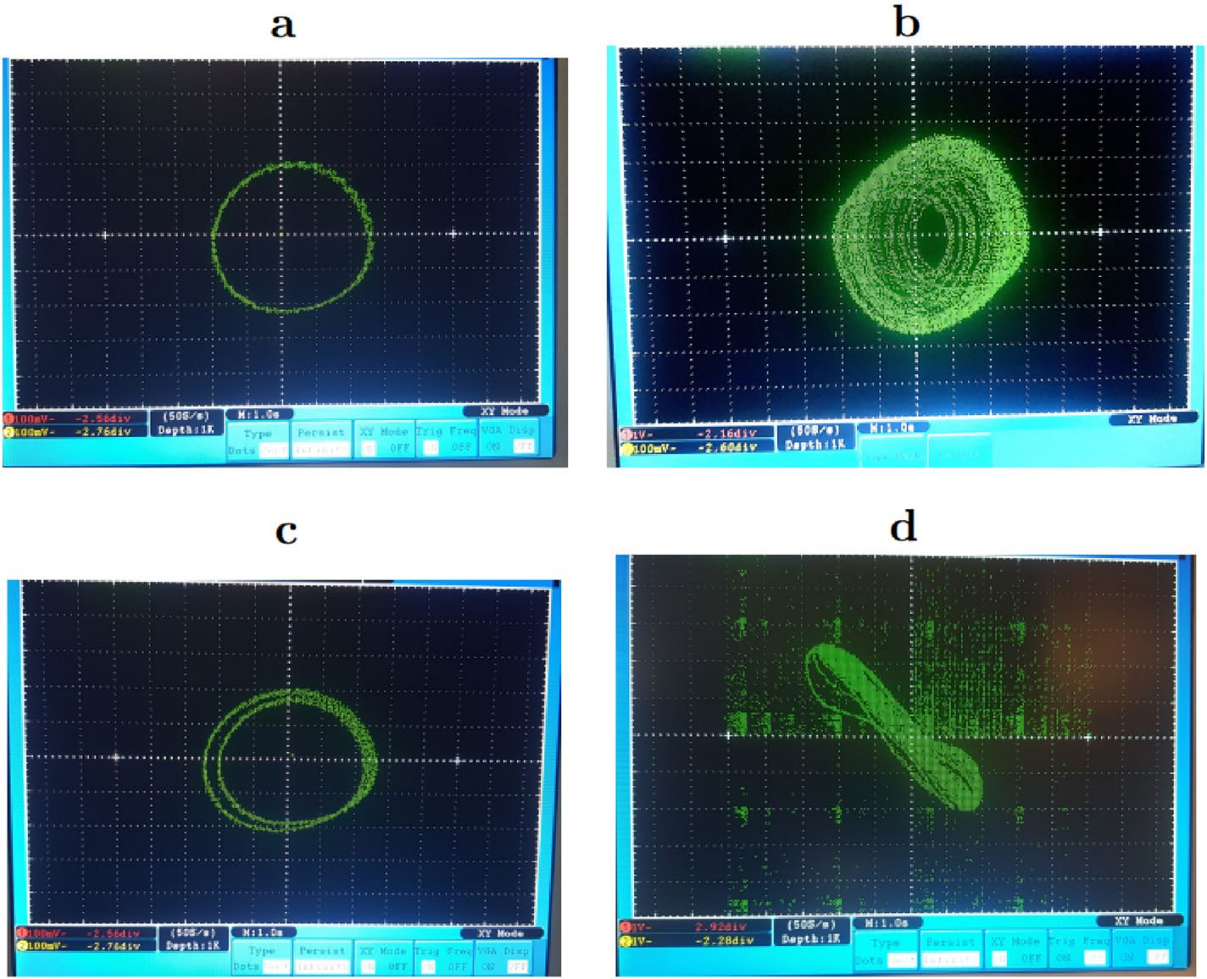
Phase portrait of *x* versus *y* obtained experimentally of the Fractional Jerk system showing route to chaos attractor when varied parameter $\beta$ (A) in (**a**) period-1 limit cycle for $\beta =0.4$, (**b**) a chaotic attractor for $\beta =0.35$. a and b are obtained with $(q_1,q_2,q_3)=(1,1,1)$. (B) in (**c**) a period-2 limit cycle attractor for $\beta =0.4$ obtained with $(q_1,q_2,q_3)=(0.96,0.97,0.97)$. (**d**) a chaotic attractor for $\beta =0.40$, obtained with $(q_1,q_2,q_3)=(0.98,1,1)$.

### Why a micro-controller implementation?

A Fractional Capacitor is a combination of several classical capacitors and resistors. Depending on the design method chosen, the number of resistors and capacitors used is large^58^. As a result, a large amount of energy is consumed. We avoid the space requirement of the device because, for an analog implementation of a third order system, three fractional capacitors need to be designed. To confirm the simplicity of a PIC implementation, observe (Fig. 5b). One notices that the experimental device contains only four main elements. There are two digital-to-analog converters (A and B), a micro-controller (C) and an oscillator of 20MHz (D). When the assembly is supplied with a 5 V voltage, oscillations are observed on the oscilloscope as shown in the Fig. 5a.

### Fractional order influences

As the numerical values, we assume the same control parameter values in agreement with the bifurcation diagram. By considering the classical case ($q_1=q_2=q_3=1$) with $\beta =0.35$, one observes a periodic dynamics, see Fig. 6a and a chaotic dynamics, see Fig. 6b respectively which are the similar attractors to those obtained under Matlab, see Fig. 3a,b ) . To prove the impact of the fractional order on the experimental solution of the problem, we proceed as in numerical simulation, i.e. by considering the value of $\beta$ for which a periodic dynamics is observed ($\beta =0.4$) and we vary the fractional orders. Thus, taking $(q_1,q_2,q_3)=(0.94,0.98,0.95)$, the Fig. 6c shows a double periodic dynamics. Still with ($\beta =0.40$) and considering $(q_1,q_2,q_3)=(0.98,1,1)$, one observes a chaotic dynamics (Fig. 6d), which was not observed for the classical case considering ($q_1=q_2=q_3=1$). The same behaviour was observed in numerical simulation under Matlab (see Fig. 6c,d), which shows that the system implemented under micro-controller is successful.

### Synchronization results

Chaos synchronization consists of oscillating two chaotic systems in a synchronized manner. So, one recognize weaker forms of synchronization, when some key characteristics of the dynamical behavior are identical, such as frequencies or amplitude. Hence, in this section, we discuss the synchronization of two fractional chaotic systems. To do this, we consider here two chaotic systems called respectively master (*m*) and slave (*s*). According to the Adaptive control method^59^, we derive the following equations:

14 $$\begin{aligned}&\qquad \qquad \qquad \qquad {\textbf {Master}}\nonumber \\&\quad \left\{ \begin{array}{lll} _0D_t^{q_1}x_m&{}=&{}y_m\\ _0D_t^{q_2}y_m &{}=&{}z_m\\ _0D_t^{q_3}z_m &{}=&{}\alpha x_m^3-x_m-y_m-\beta z_m \end{array}\right. \end{aligned}$$

15 $$\begin{aligned}&\qquad \qquad \qquad \qquad {\textbf {Slave}}\nonumber \\&\quad \left\{ \begin{array}{lll} _0D_t^{q_1}x_s &{}=&{}y_s+u_1\\ _0D_t^{q_2}y_s &{}=&{}z_s+u_2\\ _0D_t^{q_3}z_s &{}=&{}\alpha x_s^3-x_s-y_s-\beta z_s+u_3 \end{array}\right. \end{aligned}$$

wherein $u_1\, , u_2$ and $u_3$ are active non-linear controls that have been added to the chaotic chaotic system (Eq. 14) to implement the synchronisation. Considering the synchronization errors as $e_1 = x_s-x_m,~ e_2 = y_s-y_m,~ e_3 = z_s-z_m,$ we derive (Eq. 16).

16 $$\begin{aligned} \left. \begin{array}{lll} u_1 &{}=&{} -e_1 k_1-e_1\\ u_2 &{}=&{} -e_2 k_2-e_3\\ u_3 &{}=&{} \beta e_1^3+3\beta e_1^2 x_m+3 \beta e_1 x_m^2+\beta x_m^3- \alpha e_3-\alpha z_m-e_3 k_3-e_1-e_2-x_m-y_m \end{array}\right. \end{aligned}$$

**Figure 7:**
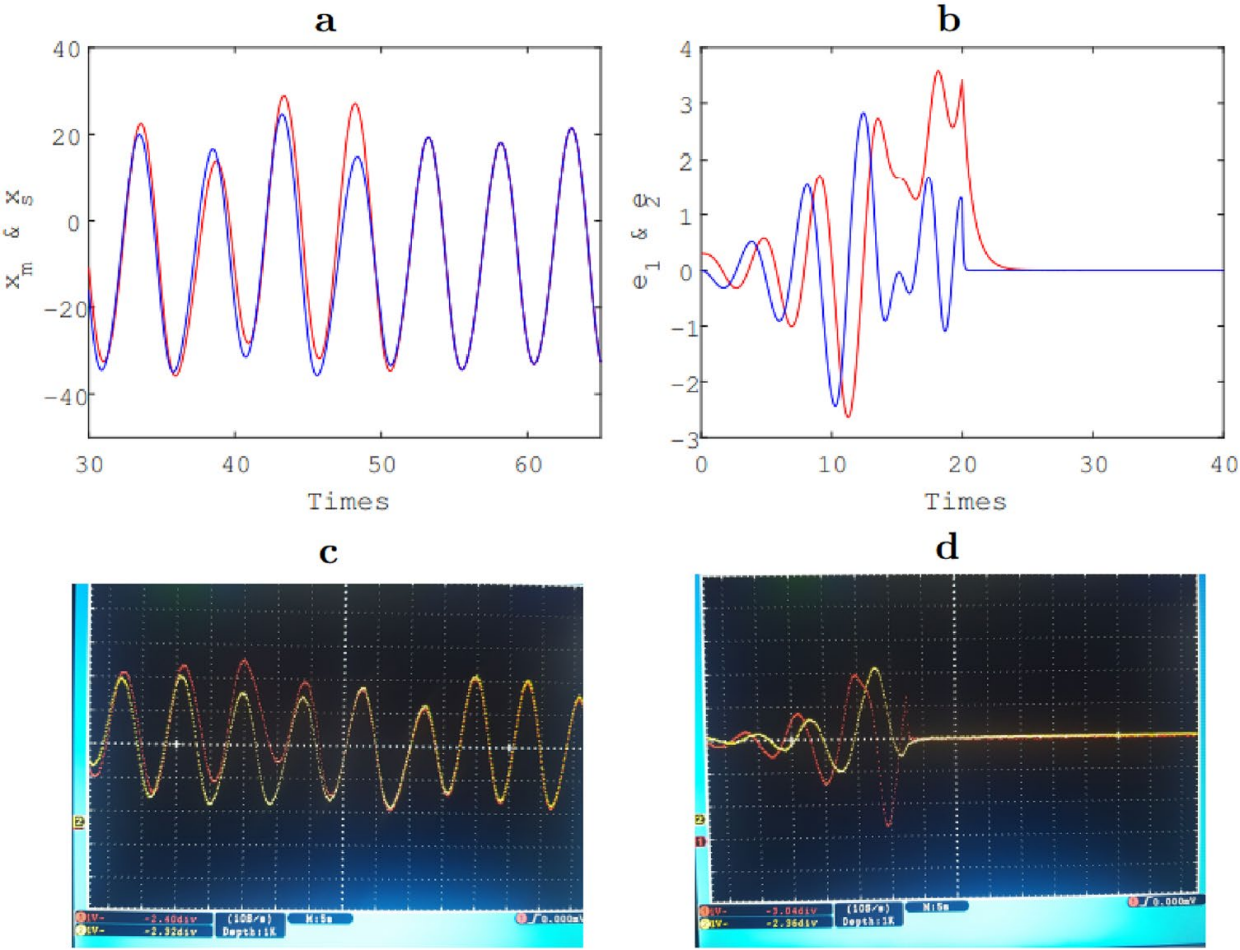
Fractionnal synchronization result: $e_1$ and $e_2$ are synchronisation error (**b**,**d**), $x_m$ is synchronized with $x_s$ (**a**,**c**). *a* and *b* are the results obtained under Matlab, *c* and *d* are the synchronization results obtained experimentally for $(q_1 , q_2 , q_3 ) = (0.98, 1, 1).$

Equations (14) and (15) are solved using the predictor-corrector method described in “Results and discussions”. It appears that, master and slave systems trajectories converge after few milliseconds as depicted on Fig. 7 where $e_1$ and $e_2$ are synchronisation error, $x_m$ is synchronized with $x_s$. Figure 7a,b are the results obtained under Matlab, Fig. 7c,d are the synchronization results obtained experimentally.

## Conclusion

In this paper, we have proposed Pic micro-controller modelling a Jerk equation in integer and fractional order domains and phase portraits were investigated numerically and experimentally. Analytic studies, Lyapunov exponents and bifurcation analysis showed that the system has three determined equilibrium points and also displays complex self-excited non-linear dynamics. It appeared from simulations and experimentations that, the fractional model of the designed circuit allows to obtain masked attractors contrarily to the classical model considering the same parameters. A study case of synchronization to overcome the extreme sensitivity of the initial conditions was investigated. As a future outcome will be the exploration under a digital development board such as the FPGA.

## Data Availability

The data that support the findings of this study are available from the corresponding author upon reasonable request.
